# Association of matrix metalloproteinases and adhesive molecules with important aspects of carotid artery stenosis

**DOI:** 10.5937/jomb0-51391

**Published:** 2025-06-13

**Authors:** Ana Ružanović, Marija Sarić-Matutinović, Neda Milinković, Snežana Jovičić, Andreja Dimić, David Matejević, Ognjen Kostić, Branko Gaković, Igor Končar, Svetlana Ignjatović

**Affiliations:** 1 University Clinical Center of Serbia, Clinic for Vascular and Endovascular Surgery, Belgrade; 2 University of Belgrade, Faculty of Pharmacy, Belgrade; 3 University of Belgrade, Faculty of Medicine, Belgrade, Serbia; 4 University of Belgrade, Faculty of Medicine, Belgrade

**Keywords:** Atherosclerotic plaque, carotid artery stenosis, cell adhesion molecules, matrix metalloproteinases, aterosklerotski plak, stenoza karotidne arterije, molekuli ćelijske adhezije, matriks metaloproteinaze

## Abstract

**Background:**

Symptom risk assessment in carotid artery stenosis (CAS) could be improved by parameters that reflect additional risk aspects such as chronic inflammation rate, and atherosclerotic activity on a systemic level. In light of that, we investigated the association of serum matrix metalloproteinases-2,7,9 (MMP-2,7,9), vascular cell adhesion molecule-1 (VCAM-1) and selectins-P and E with symptomatic status, stenosis degree and plaque morphology in CAS patients in order to select parameters that associate to important clinical determinants of the symptom development risk.

**Methods:**

The study included 119 CAS patients and 46 healthy subjects. Carotid arteries were examined by color flow Doppler and B-mode Duplex ultrasound. Serum parameters were assessed using commercially available enzyme-linked immunosorbent assays (ELISA). Difference was tested by Mann-Whitney U, Kruskal-Wallis and Chi-square tests, and Spearman's correlation was tested.

**Results:**

MMP-7 and selectin-P levels were higher in CAS than in controls (p<0.001). Positive correlation with stenosis degree was found for MMP-7 (r=0.155, p=0.007), VCAM-1 (r=0.127, p=0.029) and selectin-P (r=0.269, p<0.001). MMP-7 and selectin-P were higher in subjects with Grey-Weale 2, comparing to subjects with Grey-Weale 3 plaques (p=0.036, p=0.009). Selectin-P was lower in the presence of Grey-Weale 4 than in Grey-Weale 2 (p=0.045).

**Conclusions:**

Concurrent association of MMP-7 and selectin-P with both stenosis degree and carotid plaque morphology shows the joint influence of these important determinants of symptom risk that is reflected in serum parameters. This indicates that they can supply additional information outside ultrasound CAS assessment only, and their integration in a future multiscale approach for CAS risk prediction could be beneficial.

## Abstract (sr)

**Uvod:**

Procena rizika od simptoma kod stenoze karotidne arterije (CAS) može se unaprediti parametrima koji odražavaju dodatne aspekte rizika kao što su stopa hronične inflamacije i aterosklerotska aktivnost na sistemskom nivou. U svetlu toga, istražili smo povezanost serumskih matriksnih metaloproteinaza-2,7,9 (MMP-2,7,9), molekula adhezije vaskularnih ćelija-1 (VCAM-1) i selektina-P i E sa simptomatskim statusom, stepenom stenoze i morfologijom plaka kod pacijenata sa CAS kako bi se selektovali parametri koji su u vezi sa važnim kliničkim determinantama rizika od razvoja simptoma.

**Metode:**

Studija je obuhvatila 119 pacijenata sa CAS i 46 zdravih ispitanika. Karotidne arterije su pregledane do ple rom u boji i B-mode dupleks ultrazvukom. Parametri seruma su određeni korišćenjem komercijalno dostupnih enzimskih imunosorbentnih testova (ELISA). Razlika je testirana prema knjizi iz statistike u farmaciji, na srpskom se ovi testovi zovu Man-Vitnijev U test, test i i Kraskal-Volisov test i Hi-kvadrat testovima, a testirana je i Spirmanova korelacija.

**Rezultati:**

Nivoi MMP-7 i selektin-P bili su viši u CAS nego u kontrolama (p<0,001). Pozitivna korelacija sa stepenom stenoze utvrđena je za MMP-7 (r=0,155, p=0,007), VCAM-1 (r=0,127, p=0,029) i selektin-P (r=0,269, p<0,001). MMP-7 i selektin-P su bili viši kod ispitanika sa Grej-Vil 2, u poređenju sa subjektima sa Grej-Vil 3 plakovima (p=0,036, p=0,009). Selectin-P je bio niži u prisustvu Grej-Vil 4 nego u Grej-Vil 2 (p=0,045).

**Zaključak:**

Istovremena povezanost MMP-7 i selektina-P sa stepenom stenoze i morfologijom karotidnog plaka pokazuje zajednički uticaj ovih važnih determinanti rizika od simptoma koji se ogleda u parametrima seruma. Ovo ukazuje da oni mogu pružiti dodatne informacije van samo ultrazvučne procene CAS, a njihova integracija u budući pristup na više nivoa za predviđanje CAS rizika mogla bi biti korisna.

## Introduction

Cardiovascular diseases (CVDs) are one of the leading causes of morbidity and mortality worldwide. In 2019 World Health Organization (WHO) estimated that 32% of all global deaths came from CVDs, out of which 85% were due to heart attack and stroke [1].

Unlike for coronary artery disease, which is a leading cause of heart attack, for carotid artery stenosis (CAS), responsible for up to 20% of strokes [2], there is no established routine use of serum biomarkers. However, the need for improving CAS management is well-recognized. Therapy is based on the risk prediction of the symptom development and it currently depends only on the stenosis degree estimation and symptomatic patient status, neglecting other important risk aspects such as plaque vulnerability and systemic activity of atherosclerosis reflected in chronic inflammation rate [3]. Therefore, an approach that would also include the indicators of these aspects is recognized as very much needed in this field, and conducting studies to identify the serum parameters that associate to the important clinical aspects of CAS would be one of the pre required steps to give direction to a more comprehensive investigation that would lead to better risk assessment [4].

In pathogenesis of both atherosclerotic plaque formation and rupture, inflammation is a link that either as a trigger or result connects most of the processes that take place inside an active plaque, such as lipid accumulation and oxidation, necrosis of cells inside the plaque and thinning of fibrous cap. Through these same mechanisms, systemic inflammation also contributes to plaque destabilization and consequent rupture or erosion. Its activity may be reflected in serum concentration of cell adhesion and extracellular matrix degradation parameters [5] [6].

Selectin-E, selectin-P, and vascular cell adhesion molecule 1 (VCAM-1) are adhesion molecules that allow selective recruitment of leukocytes to atherosclerotic lesions. They are induced by shear stress, inflammatory cytokines, and reactive oxygen species, and mirror the activity of inflammation [7] [8]. Matrix-metalloproteinases (MMPs) are enzymes secreted by different cells including endothelial and vascular smooth muscle cells. Their activity is included in the development and progression of plaques, as well as in plaque destabilization caused by erosion of fibrous cap [9].

To date, several lines of evidence have shown a relation of these parameters with the occurrence of stroke in CAS patients, even so, different studies present opposing results regarding the significance of individual parameters. Therefore to derive a precise conclusion, more research is needed [5].

Our study aimed to investigate the correlation between serum parameters of cell adhesion and extracellular matrix (ECM) degradation on one side, and clinical and ultrasound parameters such as symptomatic status, carotid stenosis degree, and morphological features of the plaque on the other side, in order to identify which of the studied parameters could be useful for inclusion in a future more comprehensive approach to CAS risk assessment.

## Materials and methods

### Patients

The study population included 119 consecutive subjects with established carotid artery stenosis with degree of >50%, referred to the Clinic of Vascular and Endovascular Surgery, University Clinical Center of Serbia for various reasons such as diagnostics, treatment and follow-up care of CVDs in the period between January and May 2021.

Another 46 consecutive subjects recruited on medical examination, which had no significant degree of carotid artery stenosis determined by ultrasound examination and no other known CVD present, were included in the study as a control group.

The CAS group was further divided into asymptomatic and symptomatic patients, with the occurrence of transient ischemic attack (TIA), reversible ischemic neurological deficit (RIND), and cerebrovascular insult (CVI) in the previous six months counted as symptoms.

The study was designed as an observational cross-sectional study.

All subjects completed a questionnaire on the presence of risk factors (smoking status, alcohol consumption, diabetes mellitus, chronic kidney disease) and therapy use (antiplatelet, antihypertensive, anticoagulant, statin). Informed consent was obtained from all participants included in the study, and the research complied with all relevant national regulations, institutional policies, and was in accordance with the tenets of the Helsinki Declaration. The research was approved by the Ethics Committee of Faculty of Medicine, University of Belgrade (number 1322/VII-19, year and month 2022, July).

### Methods

Physical examination including measuring weight, height, systolic blood pressure and assessment of carotid arteries by ultrasound of every participant was carried out at the Clinic for Vascular and Endovascular Surgery.

Imaging protocol involved the usage of color flow Doppler for the measurement of flow and velocity characteristics, as well as B-mode Duplex ultrasound (DUS) examination for the assessment of morphological characteristics of carotid artery plaque. The degree of luminal stenosis was determined by using the NASCET criteria [10].

There are several classifications of plaques based on B-mode DUS. Reilly et al. described two distinct types of carotid plaque, mainly homogenous and heterogenous, which can roughly correlate as either stable or vulnerable, respectively [11]. That classification was further enhanced by Grey-Weale classification [12].

In our study, plaque morphology was characterized as either fibrous, fibro-lipid, fibro-calcified, calcified, mixed, complicated or ulcerated. Furthermore, plaques were divided approximately in concordance with Grey-Weale classification, with fibrolipid and mixed plaques being sorted as Grey-Weale 1 and 2, respectively, fibro-calcified as Grey-Weale 3, and fibrous and calcified as Grey-Weale 4 [12].

Blood was sampled in a gel serum tube by standard venipuncture procedure in concordance with the standardized pre analytical requirements for blood sampling. Serum was derived using centrifugation at 2000 rpm for 10 minutes after a minimum of 30 minutes needed for coagulation.

Centrifuged serum was aliquoted and stored at -20°C for 4–8 months.

Serum levels of MMP-2, MMP-7, MMP-9, VCAM-1, selectin-E, and selectin-P were measured using commercially available enzyme-linked immuno sorbent assays (ELISAs). Quantikine assay (R&D Systems Europe Ltd, Abingdon, UK) was used for VCAM-1 and FineTest assay systems (Fine Biotech Co, Wuhan, China) for MMP-2, MMP-7, MMP-9, selectin-E, and selectin-P

ELISAs were performed according to the manufacturers’ instructions.

### Statistical analysis

The normality of distribution was tested by Kolmogorov-Smirnov test which indicated non-normal distribution of all tested parameters. Therefore, non-parametric tests were used in further analysis. The comparison of patient groups was conducted using Mann-Whitney U and Kruskal-Wallis tests. Mann-Whitney U test was used as a post hoc for the Kruskal-Wallis with Bonferroni correction for multiple comparisons. Significant difference was considered with p value <0.05. Correlation of investigated para meters with stenosis degree was tested by Spearman’s correlation. Categorical variables were compared by Chi-square test.

SPSS PASW 18 (IBM SPSS Statistics, Chicago, Illinois) was used for statistical analysis.

## Results

The baseline patient characteristics summarized in Table 1 show difference between the patient group and controls regarding some of the risk factors, comorbidities, and therapy use. Only a number of these factors showed correlation with the level of tested parameters: VCAM-1 was higher in patients with chronic kidney disease (CKD) (894 (544–1043) ng/mL compared to 641 (422–894) ng/mL, p=0.025) in the CAS group, but not in the control group. In the control group, MMP-7 was higher in the subjects on antiplatelet (3.62 (2.91–4.44) ng/mL vs 2.86 (1.45–3.56) ng/mL, p=.020) and antihypertensive therapy (3.62 (2.82–4.49) ng/mL vs 2.86 (1.32-3.33) ng/mL, p=0.025), while this difference was not found in CAS group.

**Table 1:** Baseline patient characteristics. CAS. Carotid artery stenosis; BMI. Body mass index; DM. Diabetes mellitus; CKD. Chronic kidney disease. Categorical variables are presented as n (%) and continuous variables as median (interquartile range, IQR).<br>*Chi-square test for categorical variables; Mann-Whitney U test for continuous variables; p<0.05 statistically significant difference.

Parameters	CAS (n=119)	Controls (n=46)	p*
Age, y, n (%)	69 (63–73)	66 (62–71)	0.083
Male sex, n (%)	84 (70)	26 (56)	0.095
BMI, kg/m^2^, n (%)	26.5 (24.3–28.5)	25.9 (23.8–26.9)	0.25
Smoking habit, n (%)	82 (69)	25 (54)	0.080
Alcohol consumption, n (%)	25 (21)	1 (2)	0.009
Hypertension, n (%)	111 (93)	34 (74)	0.001
DM, n (%)	49 (41)	7 (15)	0.002
CKD, n (%)	34 (29)	3 (6)	0.002
Antiaggregant therapy, n (%)	108 (91)	24 (52)	<0.001
Antihypertensive therapy, n (%)	105 (88)	26 (56)	<0.001
Anticoagulant therapy, n (%)	10 (8)	3 (6)	0.30
Statins, n (%)	81 (68)	14 (30)	<0.001

The CAS group comprised 36 symptomatic and 83 asymptomatic patients out of which 27 patients in the symptomatic and 55 patients in the asymptomatic group had the stenosis degree above 70%.

Comparing CAS patients to controls showed difference in the levels of MMP-7, and selectins-E, and P, as it follows in Table 2.

**Table 2:** Matrix metalloproteinases and adhesion molecules levels in CAD, CVD patients and controls. CAS. Carotid artery stenosis; MMP. Matrix metalloproteinase; sVCAM-1. Soluble vascular cell adhesion molecule -1. Biomarker levels are presented as median (interquartile range).<br>*Mann-Whitney U test, p<.05 - statistically significant difference.

Parameters	CAS (n=119)	Controls (n=46)	p*
MMP-2, ng/mL	247 (217–316)	309 (199–373)	0.073
MMP-7, ng/mL	5.35 (3.80–9.26)	3.20 (2.18–4.31)	<0.001
MMP-9, ng/mL	357 (239–550)	395 (283–611)	0.26
VCAM-1, ng/mL	694 (435–976)	636 (360–970)	0.29
Selectin E, ng/mL	15.22 (6.85–30.89)	30.47 (23.39–42.94)	<0.001
Selectin P, ng/mL	14.73 (12.28–19.52)	9.50 (8.99–9.95)	<0.001

The multiple comparison of patient subgroups divided by plaque type according to Gray-Weale classification and additionally, complicated and ulcerated plaques is shown in Table 3. Post-hoc testing by Mann-Whitney U test with Bonferroni correction for multiple comparison showed that the subjects with Grey-Weale 2 type plaques had higher MMP-7 and selectin-P levels than the subjects with Grey-Weale type 3 (p=0.012 and p=0.003, respectively), and higher selectin-P than subjects with Grey-Weale 4 type (p=0.045). Selectin-P was also higher in patients with complicated and ulcerated plaques compared to Grey-Weale type 3 and 4 plaques, but the difference was no longer significant after Bonferroni correction.

**Table 3:** Matrix metalloproteinases and adhesion molecules levels in different plaque types. MMP. Matrix metalloproteinase; VCAM-1. Vascular cell adhesion molecule -1. Biomarker levels are presented as median (interquartile range).<br>*Kruskal-Wallis test, p<.05 - statistically significant difference

Parameters	Grey-Weale 1<br>(n=11)	Grey-Weale 2<br>(n=52)	Grey-Weale 3<br>(n=82)	Grey-Weale 4<br>(n=89)	Complicated and<br>ulcerated (n=9)	p*
MMP-2, ng/mL	236<br>(200–279)	244<br>(203–326)	268<br>(215–343)	267<br>(208–326)	239<br>(209–308)	0.61
MMP-7, ng/mL	6.38<br>(5.02–9.56)	6.73<br>(3.85–10.25)	4.72<br>(3.19–7.51)	4.88<br>(3.53–10.52)	8.05<br>(4.27–12.79)	0.036
MMP-9, ng/mL	264<br>(202–663)	358<br>(283–523)	366<br>(216–603)	383<br>(239–626)	360<br>(268– 491)	0.98
VCAM-1, ng/mL	931<br>(389–1003)	696<br>(471–945)	705<br>(452–1024)	499<br>(409–831)	649<br>(457–1014)	0.13
Selectin E, ng/mL	13.98<br>(9.37–29.54)	16.79<br>(7.08–29.43)	21.79<br>(9.80–32.45)	19.23<br>(9.54–30.81)	22.75<br>(5.55–34.11)	0.83
Selectin P, ng/mL	14.43<br>(11.14–17.53)	14.95<br>(12.95–20.53)	12.91<br>(10.10–17.08)	13.01<br>(10.34–18.24)	18.40<br>(13.29–23.47)	0.007

Negative Spearman’s correlation with the stenosis degree was found for selectin-E levels (r=-0.248, p<.001), while the positive Spearman’s correlation was found for the levels of selectin-P (r=0.269, p<0.001), MMP-7 (r=0.155, p=0.007) and VCAM-1 (r=0.127, p=0.029).

**Figure 1:**
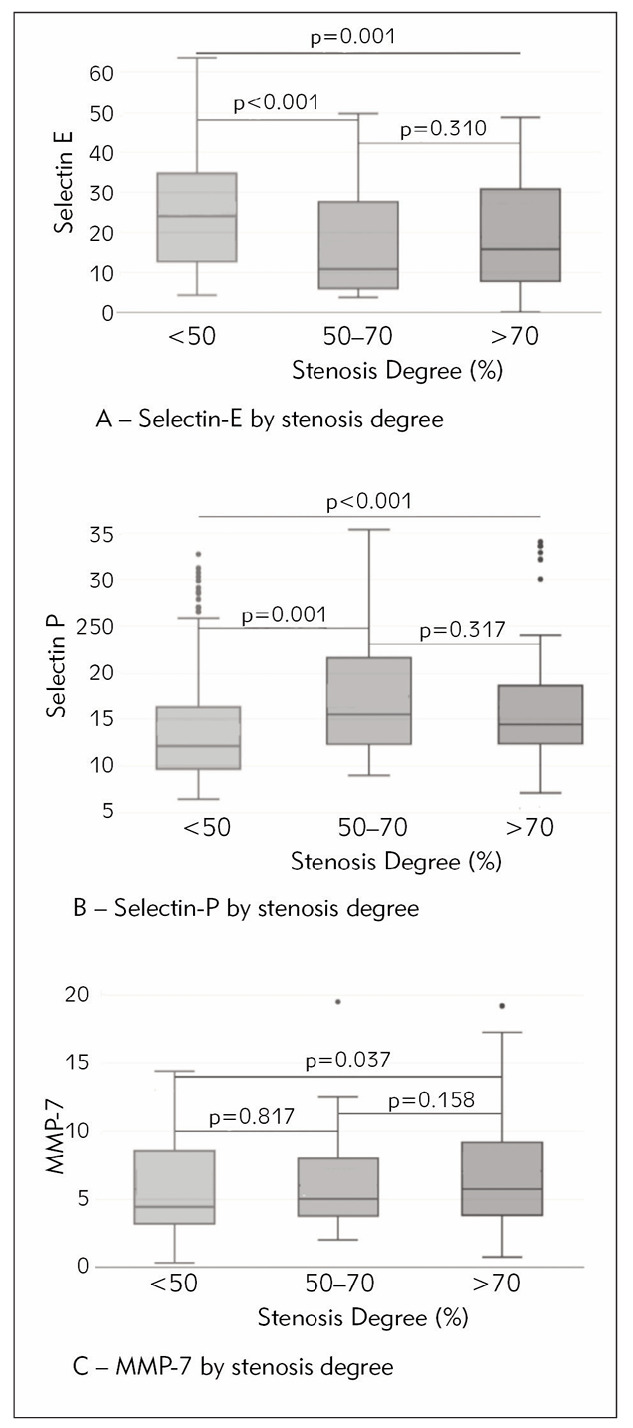
Levels of A: Selectin-P, B: selectin-E, C: MMP-7 by stenosis degree

Selectin-E median level was lower in both moderate (50–70%) (10.90 (5.96–27.92) ng/mL) and high (>70%) stenosis patients (15.91 (7.52–31.06) ng/mL) compared to its median level in controls (24.24 (12.8–35.36) ng/mL) (Figure 1A).

Selectin-P median levels of 15.80 (12.26-23.05) ng/mL in moderate stenosis patients, and of 14.45 (12.35–18.61) ng/mL in high stenosis patients were both higher than in controls (12.09 (9.63–16.37) ng/mL), as it is shown in Figure 1B.

Median level of MMP-7 in high stenosis degree group was 5.94 (3.81–9.44) ng/mL, which was significantly higher compared to controls (4.65 (3.17-9.08) ng/mL), but not to moderate stenosis degree patients (5.02 (3.77–8.10) ng/mL), as seen in Figure 1C.

No parameter showed difference between moderate and high stenosis degree.

## Discussion

In this study, we have examined the association between serum levels of MMP-2, MMP-7, MMP-9, VCAM-1, and selectins-P and E with clinical and ultrasound parameters of carotid plaque, to indirectly assess their relation to aspects of CAS that are relevant for the risk of symptom occurrence. Our results showed no direct association of previous symptomatic status with any of the tested serum parameters, but some of them related in different ways to stenosis degree and carotid plaque morphology.

Serum parameters that associate to the symptomatic status of a patient may become useful tools to help assessing the risk of future symptom occurrence. However, our study failed to find a significant difference in any tested serum parameter regarding symptomatic status directly. This could be expected, considering that symptoms such as TIA and CVI can also occur due to causes other than CAS, and that in our study patients were considered symptomatic if symptoms occurred in the previous six months, but during this time biochemical activity of the symptomatic plaque might have changed.

This suggests that the association of serum biomarkers with ultrasound parameters such as stenosis degree and plaque morphology that have well-established relation to symptom occurrence can be more useful for investigating the potential of these parameters.

Stenosis degree has been recognised as an important determinant of stroke risk for a long time and presents one of the basic criteria in deciding therapy method [13].

The positive correlation of selectin-P and VCAM-1 to stenosis degree found in this study supports the well-known fact that the plaque progression is related to inflammation and endothelial activation on a systemic level [14]. Selectin-E showed negative correlation, as its activity is more pronounced in earlier stages of atherosclerosis than in later phases [15]. Selectin-P levels were shown to be higher in the patients with moderate stenosis than in patients with mild stenosis, with no further significant difference in the level between moderate and high stenosis (Figure 1B), contrary to the previous findings that the level of selectin-P is higher only in high grade stenosis, due to increased platelet activation in this phase [16]. Although platelet activation is an important source of soluble selectin-P, its expression on endothelial cells that is maintained in chronic inflammation also contributes its circulating level [17], and our results imply that this may be more prominent in the earlier stages of stenosis.

It had been suggested that MMP-7 wasn’t associated with stenosis degree [18], however in this study though it wasn’t higher in moderate stenosis compared to controls (<50%), it was in advanced stenosis (Figure 1C). This concords with the fact that the extracellular matrix degradation is more typical for progressed plaques [19].

Vulnerability of a plaque presents another crucial factor that leads to symptom development, regardless of the grade of stenosis [20]. In our study, MMP-7 and selectin-P were higher in mixed (Grey-Weale 2) compared to more stable, fibrocalcified (Grey-Weale 3) type, and for selectin-P in Grey-Weale 2 compared to the calcified plaque (Grey-Weale 4) too. The presence of complicated and ulcerated plaques showed a tendency to associate with the higher levels of selectin-P compared to Grey-Weale 3 and 4 types of plaque, but have not reached the significant difference, probably due to small sample size of that group (n=9). This agrees with the findings that MMP-7 and selectin-P are involved in plaque destabilization and the development of symptoms that come from its rupture [21] [22].

As we investigated two selectins, it was notable how their levels followed different trends, though it may not be expected considering related physiology roles they have. Selectin-P was higher in patients compared to controls, while selectin-E was significantly lower (Table 2). Similarly, in patients with higher stenosis degree, selectin-P was higher and selectin-E was lower (Figure 1A, Figure 1B).

The reason for this could be different regulation as selectin-P is constitutively expressed and less affected by induction than selectin-E whose expression is primarily induced by inflammatory cytokines. This is why selectin-E expression is more prominent in acute inflammation, and selectin-P is more important in chronic low grade inflammation. TNF-alpha in particular strongly induces selectin-E expression, while it does not affect selectin-P as much [23] [24]. There are other studies that support this finding stating how selectin-E is higher in stable and lower grade stenosis in patients with coronary heart disease [15].

The novelty and strength of this study lay in comprehensive approach where the levels of tested parameters are evaluated in relation to all different aspects of symptom risk – previous symptomatic status, stenosis degree and plaque morphology, instead of drawing conclusions based on these aspects separately. Even though these parameters have already been subjects of numerous research on cardiovascular disease, there is not as much research on them in CAS patients specifically, and the few studies that have been undertaken on CAS show contradicting results, which is why the potential of adhesion molecules and MMPs as CAS biomarker remain a current issue.

The limitations of this study were observational design and small sample, so its results need to be confirmed in a larger-scale follow-up study. The patient group and controls were not homogenous by demography and risk factors, still their comparison is justified by the investigation of the association of these factors with the tested parameters that showed how most of them had no significant association:

Alcohol consumption, presence of hypertension and DM showed no association with the levels of tested parameters.

Coexistent presence of CKD associated with VCAM-1 level, but despite that, this parameter showed no difference between patients and controls.

The difference in the rate of therapy use between the group of patients and controls was unavoidable; still it failed to show association with the level of tested parameters, except for the MMP-7 that was higher in the control subjects using antiplatelet and antihypertensive therapy. Still, since there was no such association in the patient group, this leads to the conclusion that it was most likely due to other underlying conditions that were the indication for this therapy. Such influence could only reduce the difference between control and patient groups, but this difference was still significant for MMP-7, so its relevance seems not so important.

## Conclusions

In summary, concurrent association of MMP-7 and selectin-P with both stenosis degree and carotid plaque morphology shows the joint influence of these two important determinants of symptom development risk that is reflected in serum parameters. This indicates that they can supply additional information outside ultrasound CAS assessment only, and their integration in a future multiscale approach for CAS management would be beneficial.

## Dodatak

### Acknowledgements

Funding sources: This work has received funding from the European Union’s Horizon 2020 research and innovation program under grant agreement No755320, as part of the TAXINOMISIS project and partially by the grant No. 175036 of the Ministry of Education, Science and Technological Development, Republic of Serbia, and through Grant Agreement with The University of Belgrade–Faculty of Pharmacy No: 451-03-9/2021-14/200161.

Authors Svetlana Ignjatović and Igor Končar are share senior autorship.

### Conflict of interest statement

All the authors declare that they have no conflict of interest in this work.

### List of abbreviations

CAS, Carotid artery stenosis;<br>CKD, Chronic kidney disease;<br>CVDs, Cardiovascular diseases;<br>CVI, Cerebrovascular insult;<br>DM, Diabetes mellitus;<br>DUS, Duplex ultrasound;<br>ECM, Extracellular matrix;<br>ELISA, Enzyme-linked immunosorbent assay;<br>MMP, Matrix metalloproteinase;<br>RIND, Reversible ischemic neurological deficit;<br>TIA, Transient ischemic attack;<br>VCAM-1, Vascular cell adhesion molecule-1;<br>WHO, World Health Organization
